# Practice-Oriented Retest Learning as the Basic Form of Cognitive Plasticity of the Aging Brain

**DOI:** 10.4061/2011/407074

**Published:** 2011-10-31

**Authors:** Lixia Yang

**Affiliations:** Department of Psychology, Ryerson University, JOR823A, 350 Victoria Street, Toronto, ON, Canada M5B 2K3

## Abstract

It has been well documented that aging is associated with declines in a variety of cognitive functions. A growing body of research shows that the age-related cognitive declines are reversible through cognitive training programs, suggesting maintained cognitive plasticity of the aging brain. Retest learning represents a basic form of cognitive plasticity. It has been consistently demonstrated for adults in young-old and old-old ages. Accumulated research indicates that retest learning is effective, robust, endurable and could occur at a more conceptual level beyond item-specific memorization. Recent studies also demonstrate promisingly broader transfer effects from retest practice of activities involving complex executive functioning to other untrained tasks. The results shed light on the development of self-guided mental exercise programs to improve cognitive performance and efficiency of the aging brain. The relevant studies were reviewed, and the findings were discussed in light of their limitations, implications, and future directions.

## 1. Introduction

In research on cognition and aging, cognitive declines among older adults have been commonly documented in both cross-sectional (e.g., [1]) and longitudinal studies (e.g., [2]). The declines tend to accelerate in the advanced ages [3–6]. The cognitive declines, in combination with physical deterioration, in senior adults make them critically vulnerable in their everyday activities and could eventually deprive them of their independence and thus diminish their quality of life. This will in turn exert burdens on their families and society. This fact has spurred a growing interest in research on cognitive plasticity (i.e., the ability to improve performance through training) in older adults. The accumulated research evidence suggests that cognitive training is effective in reducing or even reversing age-related declines in target abilities that are vulnerable to aging-associated declines (for comprehensive reviews, see [7–10]). 

A large body of cognitive training studies has focused on directly teaching older adults strategies, including mnemonic techniques (for meta-analysis, see [11]), cognitive strategies facilitating performance on psychometric tests such as those for reasoning or spatial orientation (e.g., [12]). Direct strategy instruction often effectively lead to performance increments in the target cognitive tests for older adults (e.g., [12–16]; for reviews, see [17]), but the performance improvement is typically specific to the tasks directly corresponding to the taught strategies [11, 18]. In addition, learning, consolidating, and applying a new cognitive strategy are usually effortful and time consuming, thus it is unclear whether older adults would continue to use the learned strategies in their life after training. It has been shown that strategy-based training benefits tend to decrease with age [19]. Oldest-old adults (i.e., primarily in their 80s) were unable to effectively apply and optimize the newly taught mnemonic technique [20]. 

Alternatively, an emerging body of studies adopted a process-specific training approach to examine the benefits of retest practice of psychometric tests of fluid ability (e.g., reasoning, speed), executive functions (e.g., updating, switching, dual-task coordination), cognitively stimulating activities (e.g., problem solving, brain teasers) on older adults' performance on the trained or other untrained cognitive tasks ([21–23]; for reviews, see [7, 17]). The process-specific training produced sizable retest learning, namely, performance improvement on the target process through retest practice on taking the test, without instructions on task-specific strategies or adaptive feedback [9, 23]. It has been postulated that retest learning is mainly driven by reactivating or refreshing old skills that decline with age but are still available in repertoires of older adults and thus represents the basic form of cognitive plasticity [23]. Retest learning has been demonstrated in both young-old and oldest-old adults [23]. Most promisingly, some recent studies reported that older adults were able to transfer these practice-oriented training gains, primarily in the domain of executive functions, to other untrained tasks (e.g., [24]), although the majority cognitive training studies reported limited and ability-specific transfer effects. 

In the past two decades, there have been a growing number of reviews on cognitive plasticity in old age. Baltes and Linderberger [17] summarized findings of 15 years of cognitive intervention research conducted by Baltes and colleagues, primarily from two projects: ADEPT (Penn State Adult Development and Enrichment Project) and PRO-ALT (Projekt Altersintellingenz). Willis [25] identified some methodological issues in behavioral intervention studies with older adults; Kramer and Willis [26] reviewed research evidence for enhancing older adults' cognitive vitality through domain-relevant experience, laboratory-based cognitive training, and aerobic fitness training; Thompson and Foth [10] reviewed various cognitive intervention programs and their effects in enhancing mental fitness in older adults; Greenwood [27] proposed a hypothesis to view functional plasticity from the perspective of adaption of the aging brains to cognitive declines. Similarly, Goh and Park [28] proposed a “scaffolding theory” to understand neuroplasticity of the aging brain; Hertzog and colleagues [7] provided a comprehensive review of research on cognitive enrichment effects, primarily in older adults, through cognitive intervention, skill development, mentally stimulating activity, physical aerobic exercise, social engagement, as well as positive attitudes and beliefs. Lövdén and colleagues [8] proposed a theoretical framework that views cognitive plasticity as sluggish and limited intrinsic capacity for reacting to a mismatch between supply and demand. Finally, Stine-Morrow and Basak [9] reviewed different variables and approaches adopted in cognitive interventions across life-span, with a focus on aging. 

Different from all these aforementioned comprehensive reviews that focus on a general coverage of various types of experience or interventions that promote cognitive functioning of older adults, the current paper zooms in and provides an in-depth and specific review on studies that examined retest learning in aging population. Different from strategy-based training paradigms, retest learning represents a basic form of plasticity because it involves minimal externally-exerted intervention, without a need for any external guidance on task-performing strategies or adaptive feedback. It is most useful to reactivate or refresh old skills that decline with age. Relative to strategy-based training that requires a systematic guidance from an external trainer, retest learning may represent a promising and cost-effective self-guided intervention practice for older adults. 

Specifically, this paper focuses on the following core questions which have been addressed in retest learning literature. (1) Does retest learning benefit older adults? (2) Is retest learning endurable in old age? (3) Is retest learning item-specific? (4) Can older adults transfer retest learning to untrained tasks? At the end, the results would be discussed in light of their limitations and implications, together with some proposed future research directions.

## 2. Does Retest Learning Benefit Older Adults?

Data from longitudinal studies revealed substantial retest effects in cognitive abilities among adults aged 18–70 (e.g., [29–32]). Echoing with these findings, a growing body of research on cognitive plasticity indicates that older adults, including those in advanced ages, also show robust retest learning effects, mostly in fluid intelligence and executive functioning.

### 2.1. Retest Learning in Fluid Intelligence

Earlier studies on retest learning mainly focused on improving performance on tasks measuring fluid intelligence or cognitive mechanics, such as speed and reasoning (e.g., [33, 34]). Hoyer et al. [34] trained 32 elderly females (mean age = 70 years) on test-related response speed through two sessions of retest practice. The results showed sizable retest learning effects (i.e., increased response speed on the trained tests), suggesting that the age-related intellectual declines in speed may mainly reflect performance declines rather than competence deficits. Hofland et al. [33] conducted a study in which 30 young-old adults (mean age = 69 years) participated in eight sessions of retest practice on two reasoning tests: figure relations and induction. The same tests with identical items were administered across sessions and participants did not receive feedback on individual performance. The results demonstrated remarkable retest learning effects, with a total performance improvement by slightly over 1 Standard Deviation (SD). 

To investigate whether the aforementioned retest learning could be extended into oldest-old adults in their 80s and over as well as whether the magnitude of retest learning is moderated by age and cognitive functioning status, Yang et al. [23] compared 34 young olds (mean age = 74, rage = 70–79) and 34 oldest olds (mean age = 84, range = 80–91) in retest learning effects on three psychometric ability domains: reasoning, speed, and attention. Each age group was evenly divided into two groups, with high or low levels of cognitive functions relative to their age norms provided in the Berlin Aging Study (BASE, [4]). Participants completed six 1-hour retest sessions, spreading over a 3-week period, that focus on self-guided practice of 6 tasks measuring reasoning, speed, and attention. The results revealed substantial retest learning effects in both age groups across all the tasks and thus provided evidence for continued cognitive plasticity in the oldest-old age. The magnitude of retest learning was moderated by both age and cognitive functioning status in most complex reasoning tasks, with differentially lower learning rates for the oldest-old and low-status groups. For the speed task (i.e., Digit Symbol), the retest learning effect was moderated by age, but not by functioning status. For the least demanding visual attention task (i.e., D2 task), neither age nor cognitive functioning moderated the retest learning magnitude. These results suggest that age- and ability-graded declines in the basic form of cognitive plasticity, indexed by retest learning rate, only occur when the training tasks or the trained skills are complex and/or challenging. Taken together, the fluid intelligence retest training studies suggest that older adults, including those in advanced ages, are able to improve their performance on fluid ability tests through retest practice on the standard psychometric tasks.

### 2.2. Retest Learning in Executive Functioning

Retest practice paradigm has also been employed in some recent studies that aimed to improve older adults' working memory and executive functions ([21, 22, 24, 35–37]). A considerable number of studies indicate that young and older adults are capable of improving effective attentional control skills through practice on dual-task performance across a couple of sessions, with a larger training benefit demonstrated in the variable-priority training condition (i.e., shifting processing priorities between tasks in the dual-task paradigm) relative to the fixed-priority training condition (i.e., always prioritizing one task over the other in the dual-task paradigm) [21, 36, 38]. In another study, Dahlin and colleagues [35] trained young and older adults on a computer-based working memory updating task (i.e., Letter Memory) through five sessions of retest practice. Both age groups showed reliable retest learning in working memory updating performance. Furthermore, Karbach and Kray [24] administered four sessions of computerized task-switching training to children, younger and older adults. All age groups showed reliable retest learning effects on performing the trained task-switching task. In another study, Li and colleagues [22] extensively trained young (ages 20–30) and older adults (ages 70–80) on a spatial n-back working memory task across 45 days for about 15 minutes each day. Both age groups showed substantial retest learning effect as indexed by the significant performance gain on the practiced task. Similarly, Buschkuehl and colleagues [39] trained a sample of 80-year-old adults (mean age = 80.0, SD = 3.3) twice weekly for 12 weeks to practice on a variety of computerized working memory tasks that requires reproducing sequences of stimuli in an adaptive way (i.e., the sequence length was continuously adjusted to the individual's working memory capacity). The results revealed significant improvement in the performance on all the trained working memory tasks. In a recent study, Wilkinson and Yang [37] examined the retest learning in inhibition with older adults (mean age = 71.05, range = 60–84 years) using a 6-session Stroop retest training paradigm. Participants practiced on the classical color word Stroop task for about 30 minutes on each session across six retest sessions. The results showed significant linear decrease in Stroop interference effect, suggesting improvement in inhibition. The learning induced from these executive functioning or working memory training studies is considered as retest learning because the training provided is practice-oriented retest aiming to improve general process efficiency without teaching any task-specific strategies. Taken together, the executive functioning training studies suggest that deliberate retest practice is effective to improve older adults' cognitive performance on working memory or executive functioning performance. 

In sum, training studies with a practice-oriented retest paradigm on fluid psychometric intelligence (such as reasoning and speed) and executive functioning (such as updating, switching, inhibition, and working memory) consistently revealed substantial improvement in older adults, suggesting that older adults reserve the basic form of cognitive plasticity.

## 3. Is Retest Learning Item Specific or General?

In most studies involving retest learning, particularly those longitudinal or training studies on fluid intelligence, participants are repeatedly tested with identical items/tasks across the sessions. It is possible that the retest learning effects may be primarily driven by item-specific effects through memorization/familiarization with specific items and/or solutions. 

It has been argued that age effects observed in longitudinal studies are composed of positive effects associated with repeated retest practice (i.e., retest effects) and negative effects associated with age (e.g., [31, 32]). However, in most conventional longitudinal studies, identical versions of tests are administered across testing occasions. It is not very clear whether the retest effects could be minimized or even eliminated by adopting alternate versions of tests on different test occasions. In an article by Salthouse and Tucker-Drob [40], three sets of short-term retest (ranging from 1 day to a few weeks) data were analyzed and compared to evaluate the contribution of short-term retest effects in the interpretation of longitudinal change. The three studies involved in this analysis included moderately large samples of adults ranging from 18 to over 80 years of age who were tested on a cognitive battery of 16 tests at two or three occasions, with either identical or alternate versions of tests administered across two successive occasions. The results indicated that although the retest effects were greatly reduced in most tasks of memory and reasoning when the alternate versions of tests were administered, retest effects remained substantial and almost equivalent in spatial visualization and perceptual speed between the alternate-version condition (i.e., different items) and the identical-version condition (i.e., same items). These findings suggest item-specific effects may differ across ability tests, with some tests (e.g., memory) being more vulnerable to item-specific memorization effects than others (e.g., speed). Nevertheless, the results indicated that retest effects could occur even in the absence of item-specific effects in adults aged between 18 to over 80 years.

To investigate whether older adults show retest learning in the absence of item-specific effects, Yang and colleagues [41] specifically examined the non-item-specific retest learning in 31 older adults (mean age = 71.10, range = 60–82 years). The item-specific effects were eliminated through administering parallel versions of the psychometric tests of reasoning, speed, and visual attention across eight retest sessions so that participants completed a brand new set of items at each single session. The analysis on the number of correct solutions showed substantial retest improvement across all the three cognitive ability domains. However, the analysis on accuracy (i.e., the proportion of correct solutions out of all attempted items) showed a linear improvement only in the reasoning domain. Echoing with the findings of a strategy-based training study [42], this result suggested increased self-discovered strategy use in completing the reasoning tasks with retest practice. The comparison of retest learning effect size between this study and a comparable study that was vulnerable to item-specific effects (i.e., with identical tasks administered across sessions) [23] suggested that only retest learning in the reasoning domain could potentially take advantage of item-specific effects. Nevertheless, it should be noted that retest learning in reasoning was still reliable even after controlling for the contribution of item-specific effects and anxiety [41], suggesting that retest learning could occur at a more conceptual level in old age.

## 4. Long-Term Maintenance of Retest Learning

In studies with strategy-based cognitive training, it has been shown that older adults not only improve their cognitive performance through cognitive training but also maintain the training-induced improvement for a long period of time, such as up to 7 years [43, 44]. Echoing with this finding, longitudinal data also estimate that it takes 7 or more years before the positive retest effects become undetectable in longitudinal changes in cognitive functioning of adults aged 18–60 years [31]. 

However, to our knowledge, only a few studies have examined the long-term maintenance of retest learning effects in cognitive plasticity studies with older adults. Li et al. [22] investigated the practice-oriented retest training for young and older adults with a spatial n-back working memory paradigm and found substantial retest effects and transfer effects to tasks requiring similar processes. Most impressively, both age groups maintained the retest improvements and transfer effects over a period of 3 months, although the maintenance of retest learning was reduced with age. In another study, Yang and Krampe [45] compared long-term maintenance of retest learning in fluid intelligence over a period of 8 months between young-old (in their 70s) and oldest-old adults (in their 80s and onwards). The results suggested that both age groups maintained about 50% of original retest learning gains over six sessions of retest practice on psychometric tests of reasoning, speed, and attention. Together with the original study [23], this study suggests that the retest learning effects in fluid intelligence are robust and can endure for at least 8 months, even in oldest-old age. Strikingly, the maintenance effect size in reasoning over 8 months (0.45 SD) roughly corresponds to the amount of age-related decline naturally occurring over 14 years (0.42 SD) and is comparable to the trainer-guided training gain (0.48 SD) across 10 sessions [13, 45]. The maintenance effect size in speed over the 8-month period (0.66 SD) is more than 4 times the amount of age-related decline naturally occurring over 2 years (0.16 SD) [13]. Recently, some research data collected in my lab suggest that old adults are able to maintain retest learning in inhibitory control, as measured with the reduced interference effects using a Stroop retest training paradigm, for up to 1 year [46]. Taken together, the limited yet accumulating empirical findings suggest that retest learning is endurable and can be maintained for a substantial period of time in old age, even in the oldest-old age.

## 5. Can Older Adults Transfer Retest Learning to Other Untrained Tasks?

In the domain of fluid ability (e.g., reasoning and speed), transfer effects from either strategy-based training or practice-oriented retest training are very limited and highly ability specific [13, 19]. Transfer, if any, usually occurs to the similar tasks that share surface features and strategies with the practiced tasks. In a study by Baltes et al. [19], a sample of 72 elderly participants (mean age = 72 years) were randomly assigned into three conditions: a tutor-guided training group received five sessions of training that focused on teaching and identifying task-relevant problem-solving strategies and providing performance feedback on two figure relations reasoning tasks; a self-guided retest training group who went through five sessions of retest practice on the same reasoning tasks but did not receive any problem-solving strategies or feedback; a no-contact control group who did not receive any types of training. All participants completed a large set of cognitive tests at the pretest and posttest sessions. In comparison to the no-contact control group, the tutor-guided training group and the self-guided retest training group demonstrated comparable transfer effects, but the transfer was largely limited to the similar figure relations reasoning tasks. The results suggest that elderly adults are capable of achieving the same degree of performance improvement on their own through retest practice as that induced through tutor-guided strategy-based training, thus strongly supported the effectiveness of retest practice as a basic form of cognition training paradigm. Nevertheless, transfer from retest practice of fluid ability tests was largely ability specific and primarily limited to trained tasks. This limited transfer effect seems to be not due to old age of participants. In a recent large-scale study with participants from a wider age range (18–60 year), a 6-week web-based online practice of tasks measuring fluid abilities, such as reasoning, planning, problem solving skills, attention, memory, and visuospatial processing, improved performance on the trained tasks but did not produce any transfer effect to other untrained tasks, despite they measure the same abilities (e.g., reasoning and memory) [47].

Retest learning studies on executive functioning produced promising yet mixed evidence in terms of transfer effects. Similar to the training studies in fluid ability, a number of studies have demonstrated near transfer effects (i.e., transfer to similar but untrained tasks) of practice with the core process, such as executive functioning or working memory performance. In the study of Dahlin et al. [35], both young and older age groups showed substantial retest learning in working memory updating performance across five sessions of retest practice. However, only young adults transferred the retest benefits to a 3-back working memory task that also required continuous updating. Most importantly, young adults showed a training-induced activity increase in the striatum for both the trained task (i.e., Letter Memory) and the transfer task (i.e., 3 back), suggesting the transfer effect was mainly mediated by the striatum. In contrast, older adults did not show transfer effects, and their striatum activation changes were restricted to the trained task. These results suggested that older adults reserve sizable retest learning in working memory updating but this learning does not help them improve performance on other tasks requiring updating. In Li et al. [22], both young and older age groups transferred the retest learning effect in performance on a spatial working memory n-back task to another more difficult spatial n-back task and to numerical n-back tasks. The practice gains and transfer effects were maintained for 3 months, though older adults showed decrements in performance relative to postpractice performance. It should be noted that these studies focused on practicing with a single component executive functioning task (e.g., updating or n-back). Transfer effects were limited and specific to the tasks that share surface structure or measure similar or the same abilities. 

Promisingly, a growing number of studies revealed broader far transfer effects (i.e., transfer to untrained tasks measuring different abilities) from the intervention programs that target at practicing/exercising fundamental core executive functioning using multiple tasks (or multiple versions of the same task) and with complex video games. In the task-switching training study by Karbach and Kray [24], all age groups, including children, younger and older adults, demonstrated sizable near transfer effects from practicing computerized task-switching tasks across four retest sessions to structurally similar tasks and significant far transfer effects to structurally dissimilar “executive” tasks and fluid intelligence. In a related light, another recent study also suggested that practicing on a complex real-time strategy video game that requires frequent shifts in component task priority for fifteen 1.5-hour sessions over a period of 4-5 weeks could improve older adults' executive control performance [48]. In addition, another study trained old-old adults with an average age of 80 years with a 3-month (twice weekly) intervention program with a focus on practicing on a set of working memory tasks requiring reproducing sequences of colored blocks or animals on a computer screen, as well as some Reaction Time (RT) tasks. In comparison to an active control group who received physical training with an eccentric bicycle ergometer, the cognitive training group showed a sizable transfer effect to other untrained working memory and even episodic memory tasks [39]. Taken together, the significant far transfer effects appear to be evident in such training programs that focus on practicing of multiple or different versions of executive functioning tasks or engaging in activities involving complex cognitive control. 

Recently, an emerging body of studies suggested that participation in cognitively stimulating activities or immersion in an engaged life style (e.g., practice with brain teasers or puzzles, spontaneous problem solving, and helping in elementary schools), even for a short period of time, could significantly enhance fluid ability, flexible thinking, executive functions, and memory performance [49–51]. For example, in the Senior Odyssey Project conducted by Stine-Morrow and colleagues [50], older adults were randomly assigned to a 20-week program designed to operationalize an engaged lifestyle through active participation in a team-based competition in creative and spontaneous problem solving. Compared to a wait-list control group, the intervention group showed modest improvement in a composite measure of general fluid ability from pretest to posttest. This far transfer effect occurred even in the absence of explicit trainer-guided training, suggesting a possibility that the improvement may be driven by enhanced self-regulation and sustained practice of multiple abilities involved in the engaged activities. Overall, these studies suggest that older adults are able to show a broad benefit on their general cognitive ability from a short-term engagement in cognitively stimulating activities. 

Taken together, relative to retest practice of fluid ability, retest practice of executive functioning or activities involving executive control produced encouraging far transfer effects. This is probably because the executive functioning training focuses on nonspecific fundamental processes underlying performance on a broad range of cognitive tasks. In support to this assumption, a large-scale study by Smith and colleagues [52] with community-dwelling older adults suggested that 8-week practice on a brain plasticity-based computerized cognitive training program designed to improve basic cognitive efficiency (i.e., speed and accuracy) of central auditory systems could be transferred to untrained standardized measures of auditory-based memory and attention. 

In addition, there has been empirical evidence suggesting that executive functioning is largely regulated by prefrontal cortex [53], a brain region that underlies general cognitive control and shows greater and earlier signs of age-related decline than most other areas of the brain [54]. Extensive practice of executive functioning or related activities may induce neural efficiency of frontal lobe and thus improve other cognitive functions sharing the overlapped brain regions. Support for this argument could be found in Dahlin et al. [35] that suggested transfer effect of working memory updating to a 3-back task in younger adults was mainly mediated by the striatum, commonly shared between the two tasks. 

Inspired by promisingly broad transfer effects emerged from retest practice of executive functions or cognitively engaged life style, the following factors/features may potentially promote transfer effects: (1) the trained tasks and the transfer tasks share some commonalities (e.g., underlying brain regions); (2) a focus on exercising fundamental functioning or improving basic cognitive efficiency (e.g., executive control, speed and accuracy); (3) engagement of cognitively stimulating activities involving executive control and promoting self-regulation (e.g., spontaneous problem solving); (4) an extensive practice schedule lasting at least for weeks.

Taken together, like most classical cognitive training studies (e.g., [13]), the retest learning in the domain of fluid ability is relatively ability specific and only benefits similar tasks that involve the same processes as those being practiced. Nevertheless, the magnitude of transfer effects is comparable between self-guided retest practice and tutor-guided strategy-based training in the reasoning domain. The limited transfer effects tend to be narrower in scope and more restricted in older than in young adults. Promisingly, retest practice on executive functioning, working memory, and cognitively engaged life style produced encouraging far transfer benefits than retest practice on fluid intelligence tasks.

## 6. Limitations, Implications, and Future Directions

The inspiring findings reviewed above should be evaluated in light of limitations commonly observed in retest learning studies. First, most of the studies involve a highly selected sample that features high-functioning and well-educated older adults, thus we need to be careful to avoid overgeneralization of the results to low-functioning older population. Second, the exact mechanisms underlying the retest improvement are still poorly understood, although previous studies shed a light on potential contributions of item-specific memorization, self-discovered strategies with practice, familiarity with testing situation, and implicit procedural learning [31, 41]. It is also possible that the mechanisms of retest learning are different across tasks or ability domains. For example, Yang et al. [41] reported that retest learning in the reasoning domain, but not in the speed and attention domains, was partially driven by item-specific memorization. More studies are in need to pinpoint the underlying factors driving retest learning in different ability domains. Third, transfer tasks were individually selected in different studies, and they may not capture the optimal transfer effects. In any event, the generally limited and task-specific transfer effects revealed in most cognitive training studies (including the retest learning ones), especially in the domain of fluid abilities, greatly constrain the application of these interventions to benefit everyday life of older adults. Finally, retest learning does not directly help with learning new strategies or skills. In the situations where learning new strategies are required (e.g., learning how to play piano), trainer-guided strategy-based training would be most effective. 

Despite the limitations, retest learning literature revealed significant and robust retest learning improvement in the performance of practiced tasks in older adults, even in oldest-old age. A growing body of studies suggests that interventions with practicing on activities engaging complex executive functioning have promising broad transfer effects to benefit older adults' general cognitive function. These findings provide empirical foundation for practical application of the development of self-guided cognitive retest programs that could be integrated into older adults' everyday life. In comparison with most trainer-guided and strategy-based training programs, retest learning program is most economic and convenient for older adults because it does not require a trainer and it does not necessarily follow a strict schedule. Older adults could do it at their convenient time and location. All they need is a well-designed program (either a computerized program or a series of booklets) that targets the complex executive functioning and cognitive efficiency (e.g., processing speed and accuracy) of central neural system. This approach has been implemented in some recent work, although their benefits on older adults' everyday functions are still unclear. For example, Schmiedek and colleagues [55] developed a web-based training program involving 100 daily practice sessions on tasks of perceptual speed, episodic memory, and working memory. The program was evidenced to be acceptable and feasible for older adults. Some community-based programs focusing on cognitively stimulating activities and engaged life style and thus have shown promising effect on enhancing older adults' general cognitive ability [49–51]. Despite retest learning does not directly help with learning of new strategies, it should serve as the comparison baseline to correctly evaluate the strategy-based cognitive training effects [9]. Finally, retest practice of complex executive functioning may promote general cognitive efficiency and thus facilitate learning of new strategies or skills. 

In light of promising broad transfer effects of retest practice on tasks heavily loaded with requirement in frontal-lobe-dependent executive control functioning, future studies should expand and explore the optimal conditions, features, and scopes of training to be most beneficial to the daily functions of older adults. This is a challenging direction given the overall limited and task-specific transfer effect revealed in most cognitive training studies, including both strategy-based or retest learning paradigm. But it is extremely meaningful because the ultimate goal of training/practice is to improve older adults' daily functioning and reduce their independence. Future studies should also be directed to the beneficial effects of life-long practice of an engaged life-style or mental exercise materials/programs in improving older adults' cognitive functioning.

## 7. Summary and Conclusions

The existing retest learning and aging literature suggests that older adults, even those in advanced ages, reserve substantial basic form of cognitive plasticity. They demonstrate sizable retest learning benefits from practicing on tasks measuring fluid ability and executive functioning. The retest learning effects are substantial, robust, and endurable and could occur at a non-item-specific conceptual level. In the domain of fluid intelligence, older adults are also capable of transferring retest learning to other ability-specific tasks, with equivalent transfer effects from self-guided retest practice and tutor-guided training. Some recent studies show promisingly broad transfer effects from retest practice of tasks engaging executive functioning. Practice of executive functioning provides a valuable and promising self-guided intervention approach for enhancing older adults' brain functions in prefrontal cortex, general cognitive functioning, and hopefully daily activities and functions.
